# Supplementary material for: The yeast mating-type switching endonuclease HO is a domesticated member of an unorthodox homing genetic element family
Source: eLife. 2020 Apr 27;9:e55336. doi: 10.7554/eLife.55336 (PMC7282813; doi:10.7554/eLife.55336)
Supplement: Supplementary file 1. [file elife-55336-supp1.docx]

**Supplementary File 1.** *T. delbrueckii* genome sequence data used in this study.

| **Strain name** | ***FBA1* region organization** | ***FBA1* gene sequence** | **NCBI accession or Bioproject number** | **Reference** |
| --- | --- | --- | --- | --- |
| CBS1146 | Shown in Fig. 1 | CBS1146 in Fig. 3 | HE616742-HE616749 | Gordon et al. (2011) |
| (type strain) |  |  |  |  |
|  |  |  |  |  |
| L09 | Shown in Fig. 1 | L09 in Fig. 3 | PRJNA623898 | This study |
| COFT1 | Same as L09 | Identical to L09 | CP027647-CP027655 | Tondini et al. (2018) |
| SRCM101298 | Same as L09 | Identical to L09 | NJII00000000 | S.H. Cho et al., published only in database |
|  |  |  |  |  |
| NRRL Y-50541 | Shown in Fig. 1 | NRRL Y-50541 in Fig. 3 | CP011778-CP011785 | Gomez-Angulo et al. (2015) |
|  |  |  |  |  |
| L16 | Shown in Fig. 1 | L16 in Fig. 3 | PRJNA623898 | This study |
|  |  |  |  |  |
| NCYC696 | Shown in Fig. 1 | NCYC696 in Fig. 3 | PRJNA623891 | NCYC / Earlham Institute |
|  |  |  |  |  |
| L18 | Shown in Fig. 1 | L18 in Fig. 3 | PRJNA623898 | This study |
|  |  |  |  |  |
| L11 | Same as L18 | L11 in Fig. 3 | PRJNA623898 | This study |
| L15 | Same as L18 | Identical to L11 | PRJNA623898 | This study |
| L19 | Same as L18 | Identical to L11 | PRJNA623898 | This study |
|  |  |  |  |  |
| L12 | Same as L18 | L12 in Fig. 3 | PRJNA623898 | This study |
| L13 | Same as L18 | Identical to L12 | PRJNA623898 | This study |
|  |  |  |  |  |
| L10 | Same as L18 | L10 in Fig. 3 | PRJNA623898 | This study |
|  |  |  |  |  |
| L20 | Same as L18 | L20 in Fig. 3 | PRJNA623898 | This study |

We analyzed genome data from 15 *T. delbrueckii* strains as listed above, including 10 newly sequenced for this study. These 15 strains have 6 different gene organizations of their *FBA1* regions as shown in Figure 1, and they have 10 different sequence variants (alleles) of the full-length *FBA1* gene as used for phylogenetic analysis in Figure 3.
