# Supplementary material for: The yeast mating-type switching endonuclease HO is a domesticated member of an unorthodox homing genetic element family
Source: eLife. 2020 Apr 27;9:e55336. doi: 10.7554/eLife.55336 (PMC7282813; doi:10.7554/eLife.55336)
Supplement: Supplementary file 2. [file elife-55336-supp2.docx]

**Supplementary File 2.** Other genome sequence data used in this study.

| **Species** | **Strain** | **NCBI accession or Bioproject number** | **Reference for genome sequence** |
| --- | --- | --- | --- |
| *Torulaspora pretoriensis* | CBS2187 | PRJNA623867 | This study |
| *Torulaspora pretoriensis* | CBS2785 | PRJNA623867 | This study |
| *Torulaspora pretoriensis* | CBS5080 | PRJNA623867 | This study |
| *Torulaspora pretoriensis* | CBS9333 | PRJNA623867 | This study |
| *Torulaspora pretoriensis* | CBS11100 | PRJNA623867 | This study |
| *Torulaspora pretoriensis* | CBS11121 | PRJNA623867 | This study |
| *Torulaspora pretoriensis* | CBS11123 | PRJNA623867 | This study |
| *Torulaspora pretoriensis* | CBS11134 | PRJNA623867 | This study |
| *Torulaspora pretoriensis* | UWOPS 83-1046.1 | PRJNA623867 | This study |
|  |  |  |  |
| *Torulaspora franciscae* | CBS2926 | PRJNA622249 | This study |
|  |  |  |  |
| *Torulaspora globosa* | CBS764 | PRJNA625704 | This study |
| *Torulaspora globosa* | CBS2947 | PRJNA625705 | This study |
|  |  |  |  |
| *Torulaspora maleeae* | CBS10694 | PPNA00000000.2 | Shen et al. (2018) |
|  |  |  |  |
| *Torulaspora microellipsoides* | CLIB830 | FYBL00000000.1 | Galeote et al. (2018) |
|  |  |  |  |
| *Zygotorulaspora mrakii* | NRRL Y-6702 | PRJNA625702 | This study |
|  |  |  |  |
| *Zygosaccharomyces rouxii* | CBS732 | CU928181.1 | Souciet et al. (2009) |
|  |  |  |  |
| *Lachancea fantastica* | CBS6924 | FJUL01000019.1 | Vakirlis et al. (2016) |
| *Lachancea lanzarotensis* | CBS12615 | CDLU01000002.1 | Sarilar et al. (2015) |
| *Lachancea meyersi* | CBS8951 | FJUM01000009.1 | Vakirlis et al. (2016) |
| *Lachancea dasiensis* | CBS10888 | FJUP01000011.1 | Vakirlis et al. (2016) |
| *Lachancea nothofagi* | CBS11611 | FJUQ01000005.1 | Vakirlis et al. (2016) |
| *Lachancea quebecensis* | CBS14138 | CZLH01000060.1 | Freel et al. (2016) |
| *Lachancea thermotolerans* | CBS6340 | CU928168.1 | Souciet et al. (2009) |
| *Lachancea waltii* | NCYC2644 | AADM01000098.1 | Kellis et al. (2004) |
| *Lachancea mirantina* | CBS11717 | FJUN01000012.1 | Vakirlis et al. (2016) |
| *Lachancea cidri* | CBS2950 | FJUT01000004.1 | Vakirlis et al. (2016) |
| *Lachancea fermentati* | CBS6772 | FJUO01000026.1 | Vakirlis et al. (2016) |
| *Lachancea kluyveri* | NRRL Y-12651 | AACE03000004.1 | Souciet et al. (2009) |
|  |  |  |  |
| *Naumovozyma castellii* | Y056 | PRJNA623732 | This study |
| *Naumovozyma castellii* | Y174 | PRJNA623732 | This study |
| *Naumovozyma castellii* | Y287 | PRJNA623732 | This study |
| *Naumovozyma castellii* | Y668 | PRJNA623732 | This study |
|  |  |  |  |
| *Kluyveromyces lactis* | CBS2359 | CR382125.1 | Dujon et al. (2004) |
| *Kluyveromyces lactis* | CBS2105 | CP042460.1 | Varela et al. (2019) |
