# Supplementary material for: The yeast mating-type switching endonuclease HO is a domesticated member of an unorthodox homing genetic element family
Source: eLife. 2020 Apr 27;9:e55336. doi: 10.7554/eLife.55336 (PMC7282813; doi:10.7554/eLife.55336)
Supplement: Transparent reporting form [file elife-55336-transrepform.docx]

***eLife’s* transparent reporting form**

We encourage authors to provide detailed information *within their submission* to facilitate the interpretation and replication of experiments. Authors can upload supporting documentation to indicate the use of appropriate reporting guidelines for health-related research (see [EQUATOR Network](http://www.equator-network.org/%20)), life science research (see the [BioSharing Information Resource](https://biosharing.org/)), or the [ARRIVE guidelines](http://www.plosbiology.org/article/info:doi/10.1371/journal.pbio.1000412) for reporting work involving animal research. Where applicable, authors should refer to any relevant reporting standards documents in this form.

If you have any questions, please consult our Journal Policies and/or contact us: [editorial@elifesciences.org](mailto:editorial@elifesciences.org).

**Sample-size estimation**

- You should state whether an appropriate sample size was computed when the study was being designed
- You should state the statistical method of sample size computation and any required assumptions
- If no explicit power analysis was used, you should describe how you decided what sample (replicate) size (number) to use

Please outline where this information can be found within the submission (e.g., sections or figure legends), or explain why this information doesn’t apply to your submission:

This information does not apply to our submission. No explicit power analysis was used. The only place in the manuscript that involves a sample size is the decision to sequence the *ade2::TdFBA1* locus from 10 independent transformations of each type in the experiment shown in Figure 2. This sample size was chosen as the maximum that we could do conveniently. The experiment involved doing 40 yeast transformations onto separate plates, 40 genomic DNA isolations and PCRs, and 80 DNA sequencing runs.

**Replicates**

- You should report how often each experiment was performed
- You should include a definition of biological versus technical replication
- The data obtained should be provided and sufficient information should be provided to indicate the number of independent biological and/or technical replicates
- If you encountered any outliers, you should describe how these were handled
- Criteria for exclusion/inclusion of data should be clearly stated
- High-throughput sequence data should be uploaded before submission, with a private link for reviewers provided (these are available from both GEO and ArrayExpress)

Please outline where this information can be found within the submission (e.g., sections or figure legends), or explain why this information doesn’t apply to your submission:

Numbers of replicates applies only to the experiment shown in Figure 2, for which we performed 10 biological replicates as described in the previous box.

This manuscript reports a large number of new genome sequences and we currently working on submission of these genomes to the NCBI database (NCBI Bioproject numbers are provided). The sequences of the *WHO1 – WHO5* genes are already in databases (gene identifiers are given in the first paragraph of Results). The sequences of *WHO6* and of the *TdFBA1-S* and *TdFBA1-R* alleles are provided in Supplementary File 3, mentioned in Methods.

**Statistical reporting**

- Statistical analysis methods should be described and justified
- Raw data should be presented in figures whenever informative to do so (typically when N per group is less than 10)
- For each experiment, you should identify the statistical tests used, exact values of N, definitions of center, methods of multiple test correction, and dispersion and precision measures (e.g., mean, median, SD, SEM, confidence intervals; and, for the major substantive results, a measure of effect size (e.g., Pearson's r, Cohen's d)
- Report exact p-values wherever possible alongside the summary statistics and 95% confidence intervals. These should be reported for all key questions and not only when the p-value is less than 0.05.

Please outline where this information can be found within the submission (e.g., sections or figure legends), or explain why this information doesn’t apply to your submission:

Again, this applies only to the experiment in Figure 2. Since the results showed evidence of cleavage in 10 out of 10 samples in the *TdFBA1-S* + pWHO6 combination, and in zero out of 30 samples in the other combinations, we did not consider it necessary to present a statistical test of significance. (P is < 0.00001 by Fisher’s exact test).

(For large datasets, or papers with a very large number of statistical tests, you may upload a single table file with tests, Ns, etc., with reference to sections in the manuscript.)

**Group allocation**

- Indicate how samples were allocated into experimental groups (in the case of clinical studies, please specify allocation to treatment method); if randomization was used, please also state if restricted randomization was applied
- Indicate if masking was used during group allocation, data collection and/or data analysis

Please outline where this information can be found within the submission (e.g., sections or figure legends), or explain why this information doesn’t apply to your submission:

This information does not apply to our submission. Samples were not allocated into groups.

**Additional data files (“source data”)**

- We encourage you to upload relevant additional data files, such as numerical data that are represented as a graph in a figure, or as a summary table
- Where provided, these should be in the most useful format, and they can be uploaded as “Source data” files linked to a main figure or table
- Include model definition files including the full list of parameters used
- Include code used for data analysis (e.g., R, MatLab)
- Avoid stating that data files are “available upon request”

Please indicate the figures or tables for which source data files have been provided:

Supplementary File S1 (mentioned on lines 710 and 716) includes the DNA sequences of all plasmids and DNA constructs used in this study.
